# PANoptosis is a prominent feature of desmoplakin cardiomyopathy

**DOI:** 10.20517/jca.2022.34

**Published:** 2023-01-01

**Authors:** Melis Olcum, Leila Rouhi, Siyang Fan, Maya M. Gonzales, Hyun-Hwan Jeong, Zhongming Zhao, Priyatansh Gurha, Ali J. Marian

**Affiliations:** 1 Center for Cardiovascular Genetics, Institute of Molecular Medicine and Department of Medicine, University of Texas Health Sciences Center at Houston, Houston, TX 77030, USA.; 2 Heart Center & Beijing Key Laboratory of Hypertension, Beijing Chaoyang Hospital, Capital Medical University, Beijing 100020, China.; 3 Center for Precision Health, School of Biomedical Informatics and School of Public Health, The University of Texas Health Science Center at Houston, Houston, TX 77030, USA.

**Keywords:** Desmoplakin cardiomyopathy, PANoptosis, apoptosis, necroptosis, pyroptosis, inflammation, fibrosis

## Abstract

**Introduction:**

Arrhythmogenic cardiomyopathy (ACM) is hereditary cardiomyopathy caused by pathogenic variants (mutations) in genes encoding the intercalated disc (ID), particularly desmosome proteins. ACM caused by mutations in the *DSP* gene encoding desmoplakin (DSP) is characterized by the prominence of cell death, myocardial fibrosis, and inflammation, and is referred to as desmoplakin cardiomyopathy.

**Aim:**

The aim of this article was to gain insight into the pathogenesis of DSP cardiomyopathy.

**Methods and Results:**

The *Dsp* gene was exclusively deleted in cardiac myocytes using tamoxifen-inducible MerCreMer (*Myh6-Mcm*^Tam^) and floxed *Dsp* (*Dsp*^F/F^) mice (*Myh6-Mcm*^Tam^:*Dsp*^F/F^). Recombination was induced upon subcutaneous injection of tamoxifen (30 mg/kg/d) for 5 days starting post-natal day 14. Survival was analyzed by Kaplan-Meier plots, cardiac function by echocardiography, arrhythmias by rhythm monitoring, and gene expression by RNA-Seq, immunoblotting, and immunofluorescence techniques. Cell death was analyzed by the TUNEL assay and the expression levels of specific markers were by RT-PCR and immunoblotting. Myocardial fibrosis was assessed by picrosirius red staining of the myocardial sections, RT-PCR, and immunoblotting. The *Myh6-Mcm*^Tam^: *Dsp*^F/F^ mice showed extensive molecular remodeling of the IDs and the differential expression of ~10,000 genes, which predicted activation of KDM5A, IRFs, and NFκB and suppression of PPARGC1A and RB1, among others in the DSP-deficient myocytes. Gene set enrichment analysis predicted activation of the TNFα/NFκB pathway, inflammation, cell death programs, and fibrosis. Analysis of cell death markers indicated PANoptosis, comprised of apoptosis (increased CASP3, CASP8, BAD and reduced BCL2), necroptosis (increased RIPK1, RIPK3, and MLKL), and pyroptosis (increased GSDMD and ASC or PYCARD) in the DSP-deficient myocytes. Transcript levels of the pro-inflammatory and pro-fibrotic genes were increased and myocardial fibrosis comprised ~25% of the myocardium in the DSP-deficient hearts. The *Myh6-Mcm*^Tam^:*Dsp*^F/F^ mice showed severe cardiac systolic dysfunction and ventricular arrhythmias, and died prematurely with a median survival rate of ~2 months.

**Conclusion:**

The findings identify PANoptosis as a prominent phenotypic feature of DSP cardiomyopathy and set the stage for delineating the specific molecular mechanisms involved in its pathogenesis. The model also provides the opportunity to test the effects of pharmacological and genetic interventions on myocardial fibrosis and cell death.

## One sentence summary

Post-natal homozygous deletion of the *Dsp* gene leads to fulminant PANoptosis and severe myocardial fibrosis, cardiac dysfunction, arrhythmias, and premature death in mice.

## Graphical Abstract

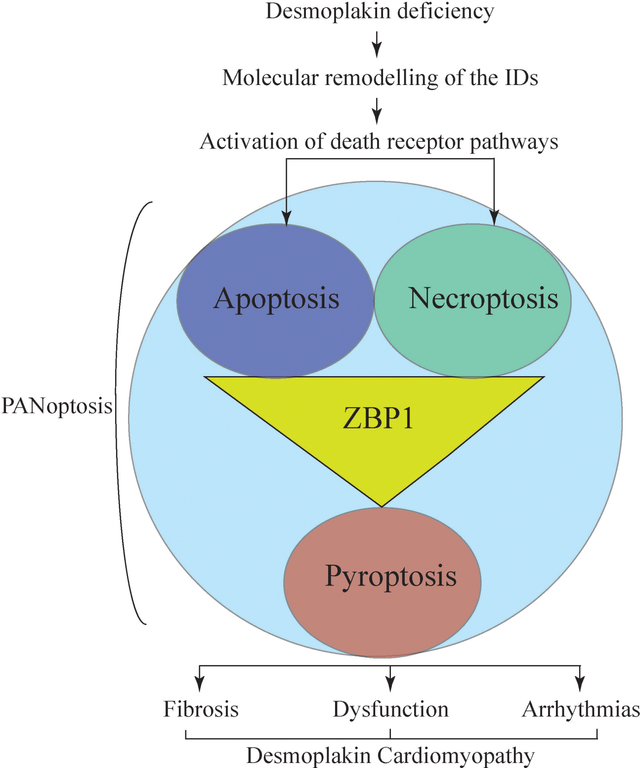

## INTRODUCTION

Arrhythmogenic cardiomyopathy (ACM) comprises a heterogeneous group of primary myocardial diseases characterized by ventricular arrhythmias, sudden cardiac death, and progressive heart failure^[1]^. The classic form of ACM shows a predilection towards the involvement of the right ventricle and is referred to as arrhythmogenic right ventricular cardiomyopathy (ARVC). The pathological hallmark of ARVC is fibrofatty infiltration of the myocardium that progresses transmurally from the epicardial surface of the right ventricles and typically involves the left ventricle in the advanced stages^[2,3]^. A subset of ACM predominantly involves the left ventricle and is referred to as arrhythmogenic left ventricular cardiomyopathy or left-dominant ACM^[4]^.

ACM is a genetically heterogeneous disease caused primarily by pathogenic variants (PVs) in genes encoding protein constituents of the intercalated discs (IDs), which are comprised of the desmosomes, adherens junction, and gap junctions. PVs in genes encoding desmosome proteins plakophilin-2 (PKP2), desmoplakin (DSP), desmoglein-2 (DSG2), desmocolin-2 (DSC2), and junction protein plakoglobin (JUP) are major causes of ACM (reviewed in^[5]^). Likewise, PVs in genes coding for the adherens junction proteins cadherin 2 (CDH2) and αT-catenin (CTNNA3) are established causes of ACM^[6,7]^. In addition, PVs in several other genes encoding proteins with a diverse array of functions, including filamin C (FLNC), phospholamban (PLN) and RNA binding motif protein 20 (RBM20), lamin A/C (LMNA) and transmembrane protein 43 (TMEM43) are also known to cause ACM^[8–12]^.

In accordance with the genetic heterogeneity, there is also considerable variability in the phenotypic expression of ACM. Consequently, there is no generalized genotype-phenotype correlation; suffice it to state that the homozygous deletion mutations in the *JUP* and *DSP* genes cause cardiocutaneous syndromes, including the Naxos disease, which are characterized by ACM, woolly hair, and keratosis^[13–15]^. Likewise, PVs in the *DSP* gene cause a distinct phenotype characterized by the prominence of myocardial cell death, fibrosis, and inflammation involving the left ventricle and resembling DCM, which is referred to as DSP cardiomyopathy^[16–20]^.

DSP is essential for embryonic development and desmosome assembly^[21,22]^. It is expressed early during embryonic development and remains stably expressed during the post-natal period^[21,22]^. The expression level of DSP is not known to change during aging or the development of other forms of cardiomyopathies or heart failure. Mutations in the *DSP* gene impair proper desmosome assembly and lead to the disassembly and disintegration of desmosomes in the advanced cases of human ACM and mouse models^[23–25]^. Consequently, DSP levels are markedly reduced in the advanced forms of ACM in humans^[24]^. Systemic and cardiac myocyte-specific homozygous deletion of the *Dsp* gene in mice impair desmosome assembly and lead to embryonic lethality^[21,22]^. To gain insight into the molecular mechanisms of DSP cardiomyopathy and to avoid embryonic lethality, we conditionally deleted both copies of the *Dsp* gene in the post-natal cardiac myocytes. The phenotype was remarkable for fulminant PANoptosis, inflammation, severe fibrosis, cardiac dysfunction, and premature death, resembling DSP cardiomyopathy. The model set the stage for delineating the molecular mechanisms involved in the pathogenesis of DSP cardiomyopathy.

## MATERIALS AND METHODS

### Regulatory approval:

The NIH Guide for the Care and Use of Laboratory Animals was followed and all animal experiments were approved by the Institutional Care and Use Committee (Protocol # AWC-21-0015).

### Post-natal conditional deletion of the *Dsp* gene specifically in cardiac myocytes:

To delete the *Dsp* gene specifically in cardiac myocytes, the *Dsp* floxed and tamoxifen-inducible *Myh6-MerCreMer* (*Myh6-Mcm*) mice were crossed and the heterozygous (*Myh6-Mcm*:*Dsp*^W/F^) and homozygous (*Myh6-Mcm*:*Dsp*^F/F^) genotype were generated [Supplementary Figure 1]. The dose of tamoxifen was 30 mg/Kg/day and was injected intra-peritoneally starting on post-natal day 14 for five consecutive days [Supplementary Figure 1]^[26–28]^. The P14 time point was selected to avoid potential untoward effects of Cre and tamoxifen on developing myocytes, as, by P14, cardiac myocytes have a negligible proliferative capacity and are post-mitotic. Littermate wild type (WT) and tamoxifen-injected *Myh6-Mcm* (*Myh6-Mcm*^Tam^) mice were used as controls.

### Genotyping:

Genotyping was performed by PCR of genomic DNA isolated from the mouse tails, as published^[22,29]^. The list of oligonucleotide primers used for the genotyping is provided in Supplementary Table 1.

### Survival analysis:

Kaplan Meier survival plots were generated in the wild type (WT), *Myh6-Mcm*^Tam^, *Myh6-Mcm*^Tam^:*Dsp*^F/F^ mice and compared using GraphPad Prism 8 software (Available from: https://www.graphpad.com/).

### Gross morphology:

Body and heart weight were measured and the ratios of the heart weight indexed to body weight 4 weeks after injection of Tam were compared among the groups.

### Echocardiography:

Echocardiography was performed as published^[28–30]^. In brief, mice were anesthetized with 1% isoflurane inhalation and left ventricular anterior wall thickness (LVAWT), posterior wall thickness (LVPWT), end-diastolic diameter (LVEDD), and end-systolic diameter (LVESD) were measured from the M-mode images using the leading-edge method. The LV fractional shortening (LVFS) and LV mass (LVM) were calculated according to a published method^[31]^. The indices of cardiac size and mass were corrected to the body weight, as the latter is a major determinant of cardiac size^[32]^.

### Electrocardiography (ECG):

Two-lead surface electrocardiogram was recorded for about an hour per mouse and analyzed, as published^[33,34]^.

### Myocardial fibrosis:

Thin myocardial sections were stained with picrosirius red and collagen volume fraction (CVF) was calculated, as published^[29,34]^. Expressions of pro-fibrotic genes were analyzed by RT-PCR, immunoblotting, and immunofluorescence staining.

### Myocardial adipocytes:

Myocardial adipocytes were detected upon staining of the thin myocardial sections with an antibody against perilipin 1 (PLIN1) after antigen retrieval, as published^[34,35]^. The secondary antibody was conjugated to Alexa 594 and the nuclei were detected upon counter-staining with 4′,6-diamidino-2-phenylindole dihydrochloride (DAPI). Approximately 20,000 DAPI-stained nuclei per genotype were examined and the percentage of cells stained positive for PLIN1 was calculated.

### Myocyte cross-sectional area (CSA):

Myocyte CSA was determined upon co-staining of thin myocardial sections with wheat germ agglutinin (WGA) and an antibody against pericentriolar material protein 1 (PCM1), which mainly marks the myocyte nuclei in the heart, as published^[29,34,36,37]^. The total area unstained with WGA and the number of PCM1-stained cells were determined using the Image J software (Available from: https://imagej.net) to calculate myocyte CSA^[35]^.

### Isolation of cardiac myocytes:

Cardiac myocytes were isolated from 4-week-old mice by the collagenase perfusion technique, as published^[24,29,30]^. In brief, mice were anesthetized with pentobarbital, the heart was excised, and the aorta was cannulated retrogradely and perfused with a collagenase digestion buffer (2.4 mg/mL) at a flow rate of 4 mL/min. The digestion reaction was stopped using a stop buffer containing 10% calf serum, 12.5 μM CaCl_2_, and 2 mM ATP. The heart was minced to free myocytes, the cell suspension was passed through a 100 μm cell strainer filter, and the myocytes were precipitated by centrifugation at 20 g. Calcium was re-introduced in a stepwise manner from 100 μM to 900 μM in the stop buffer. The isolated cardiac myocytes were suspended either in a Qiazol reagent for RNA extraction or in a protein extraction buffer for immunoblotting.

### Cell death programs:

Apoptosis was detected by the terminal deoxynucleotidyl transferase dUTP nick end labeling (TUNEL) assay using the In-Situ cell death detection Fluorescein kit, as published^[29,34]^. Approximately 20,000 DAPI-stained nuclear per mouse heart were examined to determine the percentage of cells that stained positive for the TUNEL assay. To identify the specific cell death programs activated in the heart, expression levels of the selected proteins in selected cell death programs were examined first in the myocardial and then cardiac myocyte protein extracts by immunoblotting.

### Immunocytochemistry and immunofluorescence:

Thin myocardial sections were stained with antibodies against the proteins of interest and signals were detected using fluorochrome-labeled antibodies or chromogenic detection^[33,35]^. Sections were counter-stained with DAPI, as described above. The list of antibodies used is shown in Supplementary Table 1.

### Immunoblotting:

Protein extracts from isolated cardiac myocytes or the whole heart were used for immunoblotting, as published^[29,30,38,39]^. In brief, 50 μg aliquots of protein extracts were loaded onto a 12% SDS polyacrylamide gel, electrophoresed, and transferred onto a nitrocellulose membrane. The protein of interest was detected by probing the membrane with a specific antibody, followed by treatment with the corresponding secondary antibody and the signal was detected using the ECL western blotting detection kit. Imaging was performed using a LI-COR (Odyssey) imaging system.

### Reverse transcription-polymerase chain reaction (RT-PCR):

The transcript levels of selected genes were quantified by RT-PCR of total RNA extracted from cardiac myocytes or myocardium using the miRNeasy Mini Kit, and treatment of the extracts with DNAse I. Approximately 1 μg of total RNA was used in the RT reaction and random primers and a high-capacity cDNA synthesis kit were used. The SYBR green or TaqMan assays were performed in duplicates and the mean values were normalized to the *Vcl* transcript levels. The ΔΔCT method was used to calculate the relative fold changes in the transcript levels compared to the WT myocytes. The primers used in the study are listed in Supplementary Table 1.

### RNA-Sequencing (RNA-Seq):

The RNA-Seq was performed in ventricular myocyte RNA, as published^[28,35]^. In brief, total RNA was extracted using the miRNeasy Mini Kit, RNA integrity was assessed using an Agilent Bioanalyzer RNA chip, and samples with an RNA Integrity Number (RIN) of > 8 were used to deplete the ribosomal RNA and construct strand-specific sequencing libraries. The latter was generated using TruSeq stranded total RNA library preparation kit. The sequencing reaction was performed on an Illumina HiSeq 4000 instrument to generate 75bp paired-end reads.

### Bioinformatics analysis of the RNA-Seq data:

The quality of the RNA sequence reads was assessed by FASTQC and gene read counts were determined after aligning them to the mouse reference genome build mm10 using the STAR program^[40]^. The GENCODE gene model was used to annotate the uniquely aligned read pairs (Available from: https://www.gencodegenes.org/mouse/). The DESeq2 statistical program in the R package was used to identify the differentially expressed genes (DEGs)^[41]^. The threshold for significance was set at a Benjamini-Hochberg false discovery rate-adjusted *P*-value (*q*-value) of < 0.01. The heat maps and volcano plots were visualized using the R studio (Available from: www.rstudio.com).

The Gene Set Enrichment Analysis (GSEA, version 2.2.3) was used to predict the dysregulated biological pathways (Available from: http://software.broadinstitute.org/gsea/). Enrichment of the Hallmark gene sets and canonical pathways from the Molecular Signature Database (MSigDB) 3.0 collection was predicted based on Normalized Enrichment Score (NES) and a *q*-value of < 0.05. The upstream regulators of gene expression were predicted using the Ingenuity Pathway Analysis software (IPA®, QIAGEN Redwood City). A *P*-value of < 0.05 for the overlap with the IPA target genes and a predicted *Z* score of < −2 or > 2 were considered significant.

### Statistical methods:

The data were analyzed for a Gaussian distribution using the Shapiro-Wilk normality test and those following normal distribution were compared by the *t*-test between the two groups or one-way ANOVA among multiple groups followed by Bonferroni pairwise comparisons. Data that departed from normality were compared by the Kruskal-Wallis test and the categorical data by Fisher’s exact or chi-square test. The ΔΔCT values were used to compare changes in the transcript levels. Kaplan-Meier survival plots were constructed and compared using the Log-rank test. Statistical analyses were performed using Graph pad Prism 8 or STAT IC, 15.1.

## RESULTS

### Deletion of the *Dsp* gene in the post-natal cardiac myocytes:

**Figure 1.**
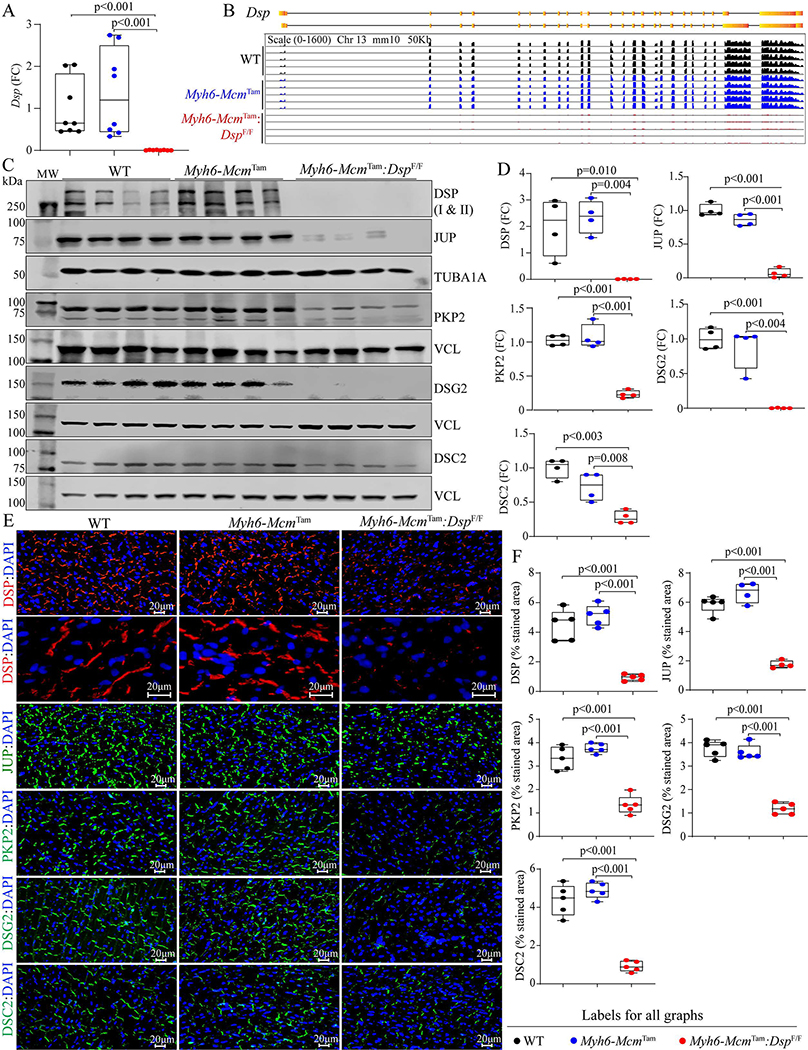
Tamoxifen-inducible Cre-mediated deletion of the *Dsp* gene in the post-natal cardiac myocytes in mice and molecular remodeling of the intercalated discs (IDs). (A) RT-PCR analysis of the *Dsp* transcript levels in cardiac myocytes isolated the wild type (WT), *Myh6-Mcm* ^Tam^, and *Myh6-Mcm*^Tam^:*Dsp*^F/F^ mice. (B) Genome browser illustration of the RNA sequencing reads mapped to each exon of the *Dsp* gene. (C) Immunoblots showing expression of selected ID proteins in WT, *Myh6-Mcm* ^Tam^, and *Myh6-Mcm*^Tam^:*Dsp*^F/F^ mice. VCL is used as a control for the loading conditions. (D) Quantitative data of selected ID proteins representing the blots shown in Panel C. Data are presented as fold change (FC). Both isoforms of DSP are included in the quantitative data. The data on the test proteins are normalized to the corresponding TUBA1A or VCL levels and are compared to the corresponding WT group throughout the manuscript. (E) Immunofluorescence panels showing expression and localization of selected ID proteins. Higher magnification panels to illustrate the localization of the DSP at the desmosomes in the genotype groups are also presented. (F) Quantitative data showing surface areas occupied by the selected ID proteins shown in Panel E.

The approach to generate *Myh6-Mcm*^Tam^:*Dsp*^W/F^ and *Myh6-Mcm*^Tam^:*Dsp*^F/F^ mice is depicted in Supplementary Figure 1. Homozygous deletion of the *Dsp* gene in the post-natal myocytes was associated with a marked reduction in the *Dsp* transcript levels (99.4 ± 0.6%) in the myocardial RNA extracts compared to the *Myh6-Mcm*^Tam^ or WT mice [Figure 1A]. The *Dsp* transcript levels were also analyzed in the RNA-Seq data upon the mapping of the *Dsp* transcript reads to the coding exons of the *Dsp* gene. The read counts were visualized using the IGV Genome Browser, which showed nearly complete depletion of the transcript reads mapped to the *Dsp* exons and excluded expression of an aberrant splice variant in the *Myh6-Mcm*^Tam^:*Dsp*^F/F^ myocyte transcripts [Figure 1B]. At the protein level, DSP was almost absent in the *Myh6-Mcm*^Tam^:*Dsp*^F/F^ cardiac protein extracts, as analyzed by immunoblotting [Figure 1C and D]. Immunofluorescence staining of the thin myocardial sections corroborated the findings, showing reduced expression and localization of DSP to the IDs, compared to the WT or *Myh6-Mcm*^Tam^ mouse hearts [Figure 1E and F].

### Molecular remodeling of the IDs:

There was a considerable molecular remodeling of the desmosomes in the *Myh6-Mcm*^Tam^:*Dsp*^F/F^ mouse hearts, indicated by the reduced expression levels of desmosome proteins JUP, PKP2, DSG2, and to a lesser extent DSC2 [Figure 1C and D]. Likewise, immunofluorescence staining of myocardial sections showed reduced localization or absence of the selected desmosome proteins at the IDs [Figure 1E and F]. At the RNA level, only transcript levels of *Jup* were modestly increased and those of *Pkp2, Dsc2, and Dsg2* were unchanged in the *Myh6-Mcm*^Tam^:*Dsp*^F/F^ mouse hearts, compared to the control groups [Supplementary Figure 2A]. Mapping of the sequence reads in the RNA-Seq data to the coding exons of the corresponding genes showed no notable differences [Supplementary Figure 2B]. In the absence of a reduction in the corresponding transcript levels, the levels of the selected ammonia protein decrease, indicating a post-transcriptional reduction of the selected desmosome proteins in the *Myh6-Mcm*^Tam^:*Dsp*^F/F^ mouse hearts.

Heterozygous deletion of the *Dsp* gene in the post-natal cardiac myocyte led to a 46.0 ± 7.6% reduction in the DSP; however, levels of DSG2, DSC2, JUP, and PKP2 were unchanged [Supplementary Figure 3A and B]. Likewise, immunofluorescence staining of thin myocardial sections detected the localization of the selected desmosome proteins to the IDs [Supplementary Figure 3C]. Moreover, transcript levels of genes encoding selected desmosome proteins, except for the *Dsp* gene, were unchanged in *Myh6-Mcm*^Tam^:*Dsp*^W/F^ mouse cardiac myocytes [Supplementary Figure 3D]. Given the modest molecular remodeling of the IDs, the *Myh6-Mcm*^Tam^:*Dsp*^W/F^ mice were not fully characterized.

### Differentially expressed genes (DEGs) and the dysregulated pathways:

**Figure 2.**
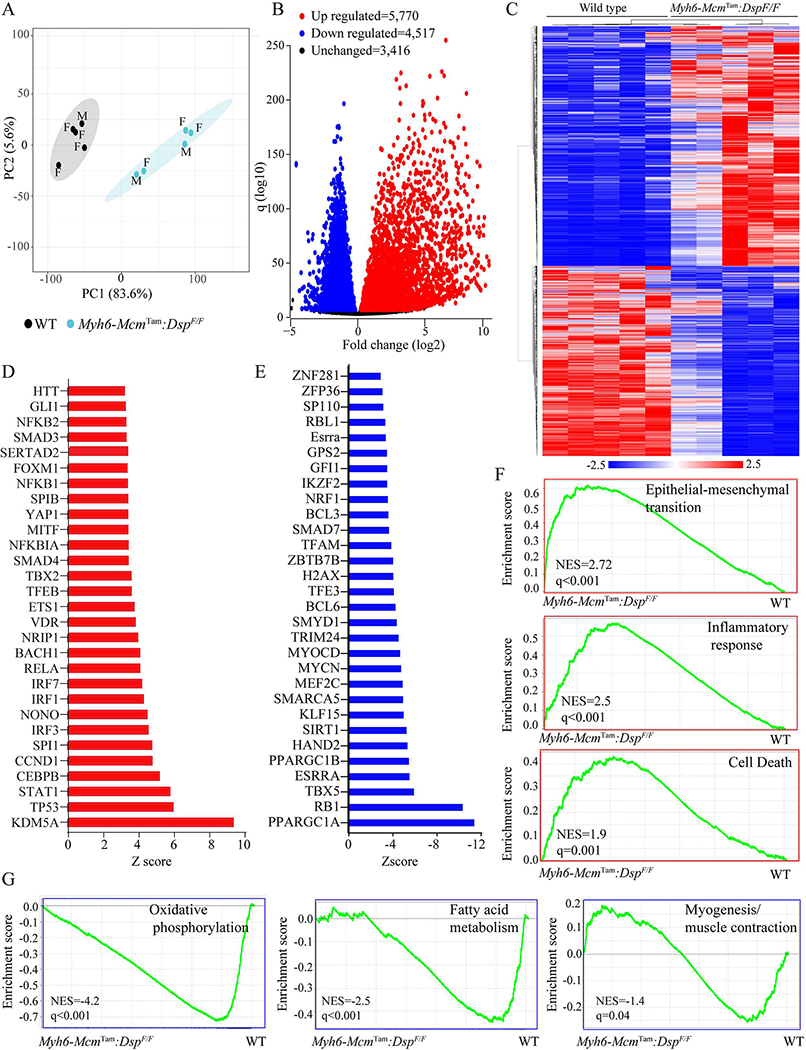
Cardiac myocyte transcripts analyzed by RNA sequencing. (A) Principal Component Analysis (PCA) of the transcripts of cardiac myocytes isolated from 4-week-old WT and *Myh6-Mcm*^Tam^:*Dsp*^F/F^ mice. Male (M) and Female (F) mice are marked. (B) Volcano plot of the differentially expressed genes (DEGs) between the WT and *Myh6-Mcm*^Tam^:*Dsp*^F/F^ myocytes. The upregulated genes are shown in red, the down-regulated ones in blue, and the unchanged ones in black. (C) Heat map of the DEGs between the WT and *Myh6-Mcm*^Tam^:*Dsp*^F/F^ myocytes. The scale is shown at the bottom of the map. (D) List of the transcriptional regulators of genes expression predicted to be activated based on the DEGs in the *Myh6-Mcm*^Tam^:*Dsp*^F/F^ myocytes. (E) List of the transcriptional regulators of genes expression predicted to be suppressed based on the DEGs in the *Myh6-Mcm*^Tam^:*Dsp*^F/F^ myocytes. (F) Gene set enrichment analysis (GSEA) maps showing enrichment of genes involved in the epithelial-mesenchymal transition (EMT), inflammatory response, and cell death in the *Myh6-Mcm*^Tam^:*Dsp*^F/F^ myocytes. (G) GSEA maps predicting suppression of oxidative phosphorylation, fatty acid metabolism, and myogenesis (muscle contraction) in the *Myh6-Mcm*^Tam^:*Dsp*^F/F^ myocytes.

Gene expression was analyzed by RNA-Seq in cardiac myocytes isolated from 4-week-old WT and *Myh6-Mcm*^Tam^:*Dsp*^F/F^ mice. A total of 346 genes (168 up-regulated and 178 down-regulated) whose transcript levels were affected by the injection of tamoxifen and activation of the Cre recombinase, as described previously, were excluded from the analysis^[28]^. Principal Component Analysis (PCA) showed distinct separation of the transcripts of WT and *Myh6-Mcm*^Tam^:*Dsp*^F/F^ cardiac myocytes [Figure 2A]. A total of ~10,300 genes were differentially expressed (*q* < 0.01), which were comprised of 4517 down-regulated and 5770 up-regulated genes. The DEGs are depicted in the volcano plots and heat map in Figure 2, Figure 2B and C. The DEGs predicted increased transcriptional activities of KDM5A, TP53, and the transcriptional regulators of inflammation, such as IRFs and NFκB, among others [Figure 2D]. Likewise, over a dozen transcriptional regulators of gene expression were predicted to be suppressed, including the PPARGC1A and RB1 [Figure 2E]. With regards to the biological pathways, the GSEA predicted activation of epithelial-mesenchymal transition (EMT), inflammatory responses, cell death apoptosis, suppression of oxidative phosphorylation and fatty acid metabolism, and to a lesser degree, myogenesis/muscle contraction [Figure 2F and G].

### Cell death programs:

Because the RNA-Seq data predicted activation of the cell death programs in the *Myh6-Mcm*^Tam^:*Dsp*^F/F^ cardiac myocytes, selected cell death programs were analyzed by the TUNEL assay and determining expression levels of the specific molecular markers.

### Apoptosis:

**Figure 3.**
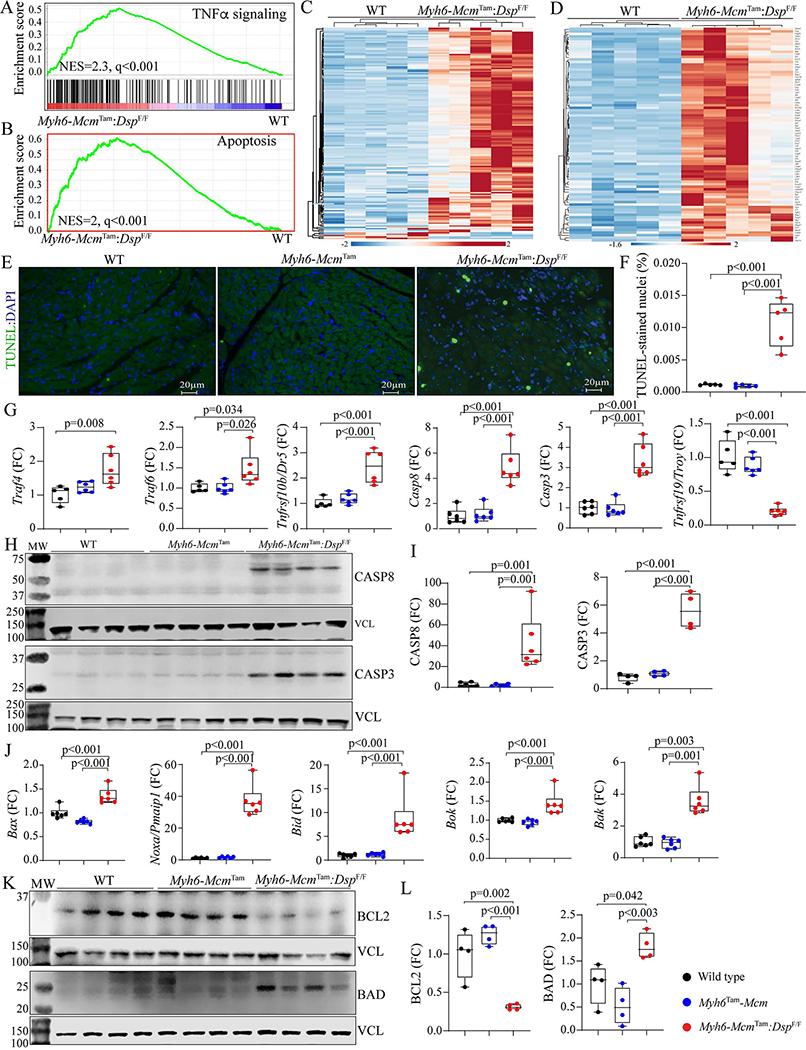
Activation of apoptosis in the *Myh6-Mcm*^Tam^:*Dsp*^F/F^ mice. (A) GSEA plot predicting activation of the TNFα signaling pathway in the *Myh6-Mcm*^Tam^:*Dsp*^F/F^ myocytes. (B) GSEA plot showing enrichment of genes involved in apoptosis in the *Myh6-Mcm*^Tam^:*Dsp*^F/F^ myocytes. (C) Heat map of the DEGs showing upregulation of most of the genes involved in TNFα signaling pathway in the *Myh6-Mcm*^Tam^:*Dsp*^F/F^ myocytes. (D) Heat map of the DEGs showing upregulation of most of the genes involved in the apoptosis in the *Myh6-Mcm*^Tam^:*Dsp*^F/F^ myocytes. (E) Thin myocardial sections stained with the TUNEL assay. (F) Quantitative data showing the percent of nuclei stained positive for the TUNEL assay in the experimental groups. (G) Dot plots showing transcript levels of selected markers of apoptosis, as detected by RT-PCR of cardiac myocyte RNA extracts. (H) Immunoblots showing expression of CASP8 and CASP3 along with that of VCL, the latter as a control for the loading condition. (I) Quantitative data representing the blots shown in panel H. (J) Immunoblots showing expression of BCL2 and BAD along with that of VCL, the latter as a control for the loading condition. (K) Quantitative data representing the blots shown in panel J.

GSEA of cardiac myocyte transcripts indicated enrichment of the genes in the TNFR-mediated cell death pathway, including apoptosis [Figure 3A and B]. Heat maps depicting the upregulation of expression of genes involved in the TNFR-mediated cell death and apoptosis pathways are depicted in Figure 3, Figure 3C and D. Apoptosis was further analyzed by the TUNEL assay, which showed a marked increased percentage of the TUNEL-positive cells in the myocardium of the *Myh6-Mcm*^Tam^:*Dsp*^F/F^ mice [Figure 3E and F]. To test for corroboration of the findings, transcript levels of several genes involved in the death receptor- and mitochondria-mediated apoptosis were analyzed in independent samples by RT-PCR, which showed upregulation of the transcript levels of dozen genes, except for the *Tnfrsf19*, which was markedly down-regulated [Figure 3G]. According to these findings, levels of CASP8 protein and its downstream target CASP3 were markedly increased in the *Myh6-Mcm*^Tam^:*Dsp*^F/F^ myocytes [Figure 3H and I].

Because CASP8 is a molecular link between death receptor-mediated (extrinsic) and mitochondrial-mediated apoptosis (intrinsic), transcript levels of several genes involved in mitochondria-mediated apoptosis were analyzed by RT-PCR, which showed increased levels of *Bax*, *Noxa* (*Pmaip1*), *Bid*, *Bok*, and *Bak* transcripts in cardiac myocytes isolated from the *Myh6-Mcm*^Tam^:*Dsp*^F/F^ mouse hearts [Figure 3J]. In agreement with the activation of the apoptotic program, levels of anti-apoptosis protein BCL2 were reduced while the level of the pro-apoptotic protein BAD was increased [Figure 3K and L]. Thus, collectively, the findings indicate activation of the death receptor- and mitochondria-mediated apoptosis.

### Necroptosis:

**Figure 4.**
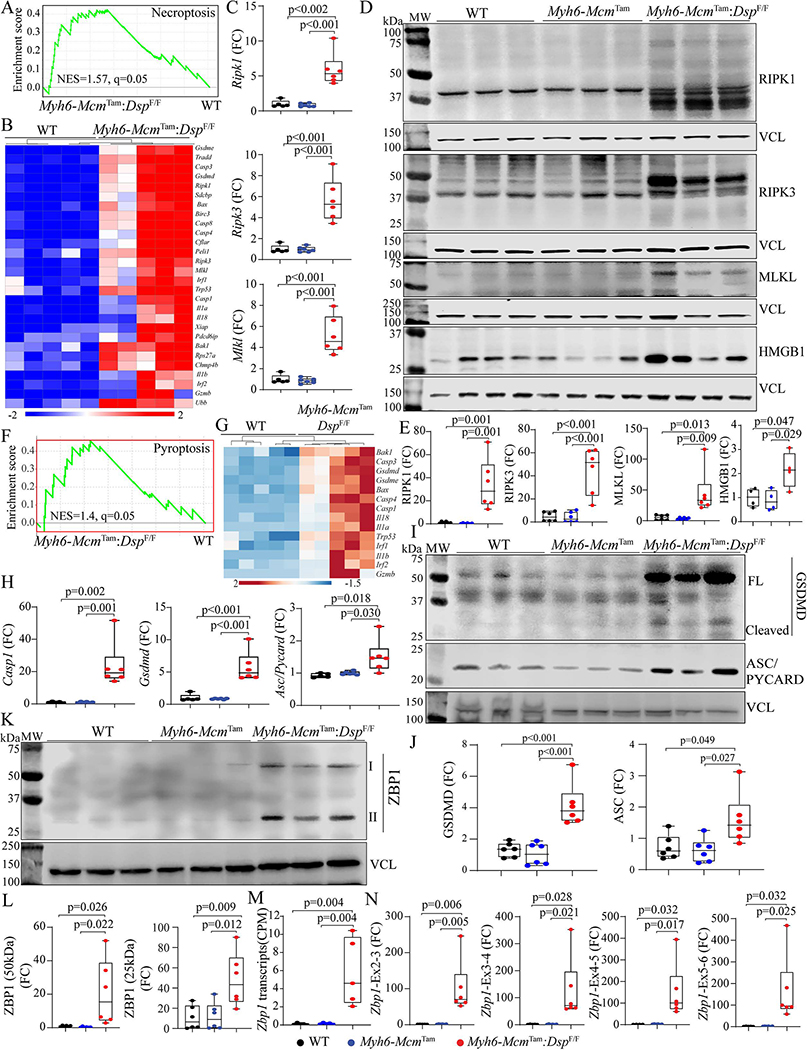
Activation of necroptosis and pyroptosis in the *Myh6-Mcm*^Tam^:*Dsp*^F/F^ mice. (A) GSEA plot showing enrichment of genes involved in necroptosis in the *Myh6-Mcm*^Tam^:*Dsp*^F/F^ cardiac myocytes. (B) A heap map of the DEGs showing upregulation of genes involved in necroptosis in the *Myh6-Mcm*^Tam^:*Dsp*^F/F^ cardiac myocytes. (C) Dot plots showing transcript levels of *Ripk1*, *Ripk3*, and *Mlkl* in the isolated cardiac myocytes, as detected by RT-PCR. (D) Immunoblots showing expression of RIPK1 (cleaved), RIPK3, MLKL, and HMGB1 in the cardiac protein extracts in the experimental groups. (E) Dot plots showing quantitative data representing the blots in panel D. (F) GSEA plot showing enrichment of genes involved in pyroptosis in the *Myh6-Mcm*^Tam^:*Dsp*^F/F^ cardiac myocytes. (G) A heap map of the DEGs showing upregulation of genes involved in pyroptosis in the *Myh6-Mcm*^Tam^:*Dsp*^F/F^ cardiac myocytes. (H) Dot plots showing transcript levels of *Casp1*, *Gsdmd*, and *Asc (Pycard)* in the isolated cardiac myocytes, as detected by RT-PCR. (I) Immunoblots showing expression of GSDMD and ASC (PYCARD) in the cardiac myocyte protein extracts in the experimental groups. (G) Dot plots showing quantitative data representing the blots in panel I. (K) Immunoblot showing expression of two isoforms of ZBP1 proteins in cardiac myocyte protein extracts. (L) Dot plots showing quantitative data representing the blots in panel K. Each ZBP1 isoform is analyzed separately. (M) *Zbp1* transcript read numbers in the cardiac myocytes RNA-Seq data presented as copy per million reads (CPM). (N) Dot plots showing transcript levels of the *Zbp1* genes, using multiple sets of oligonucleotide primers to detect its isoforms.

Because activation of the TNFR pathway also triggers programmed necrosis or necroptosis, the RNA-Seq data were analyzed by GSEA for the enrichment of genes involved in necrosis, which predicted activation [Figure 4A]. A heat map of genes involved in necroptosis shows upregulation of their expression in the *Myh6-Mcm*^Tam^:*Dsp*^F/F^ myocytes [Figure 4B]. In accordance with the RNA-Seq data, transcript levels of *Ripk1*, *Ripk3*, and *Mlkl*, the key molecules in the necroptosis pathway, were increased [Figure 4C]. Moreover, levels of RIPK1, cleaved RIPK3, and the downstream effector of the latter MLKL proteins, as well as HMGB1, were increased in *Myh6-Mcm*^Tam^:*Dsp*^F/F^ myocytes [Figure 4D and E].

### Pyroptosis:

GSEA of the RNA-Seq data suggested activation of cell death by inflammation, i.e., pyroptosis [Figure 4F]. A heat map of selected DEGs involved in pyroptosis showed upregulation of their expression, as depicted in Figure 4G. Transcript levels of *Casp1*, *Gsdmd*, and *Asc* (*Pycard*) involved in pyroptosis were markedly increased, as detected by RT-PCR in independent samples in the *Myh6-Mcm*^Tam^:*Dsp*^F/F^ myocytes [Figure 4H]. Finally, analysis of expression levels of selected proteins involved in pyroptosis showed marked upregulation of GSDMD and ASC (PYCARD) in the *Myh6-Mcm*^Tam^:*Dsp*^F/F^ myocytes [Figure 4I and J].

### Upregulation of the PANoptosis regulator Z DNA/RNA binding protein 1 (ZBP1):

Given the activation of the cell death programs by apoptosis, necroptosis, and pyroptosis, which are collectively referred to as PANoptosis, expression of the ZBP1 protein, which is considered a “master” regulator of PANoptosis, was analyzed in cardiac myocyte protein extracts by immunoblotting^[42]^. Levels of both isoforms (50 and 25 kDa) of the ZBP1 protein were markedly increased in *Myh6-Mcm*^Tam^:*Dsp*^F/F^ myocytes [Figure 4K and L]. Because ZBP1 expression is transcriptionally regulated, transcript levels of the *Zbp1* gene were analyzed in the RNA-Seq, which showed an ~4-fold increase in the *Myh6-Mcm*^Tam^:*Dsp*^F/F^ myocytes [Figure 4M]. The finding was corroborated by RT-PCR using multiple sets of primers to encompass alternative splicing of the *Zbp1* gene in independent samples, which showed marked upregulations of the transcripts [Figure 4N].

### Myocardial fibrosis:

**Figure 5.**
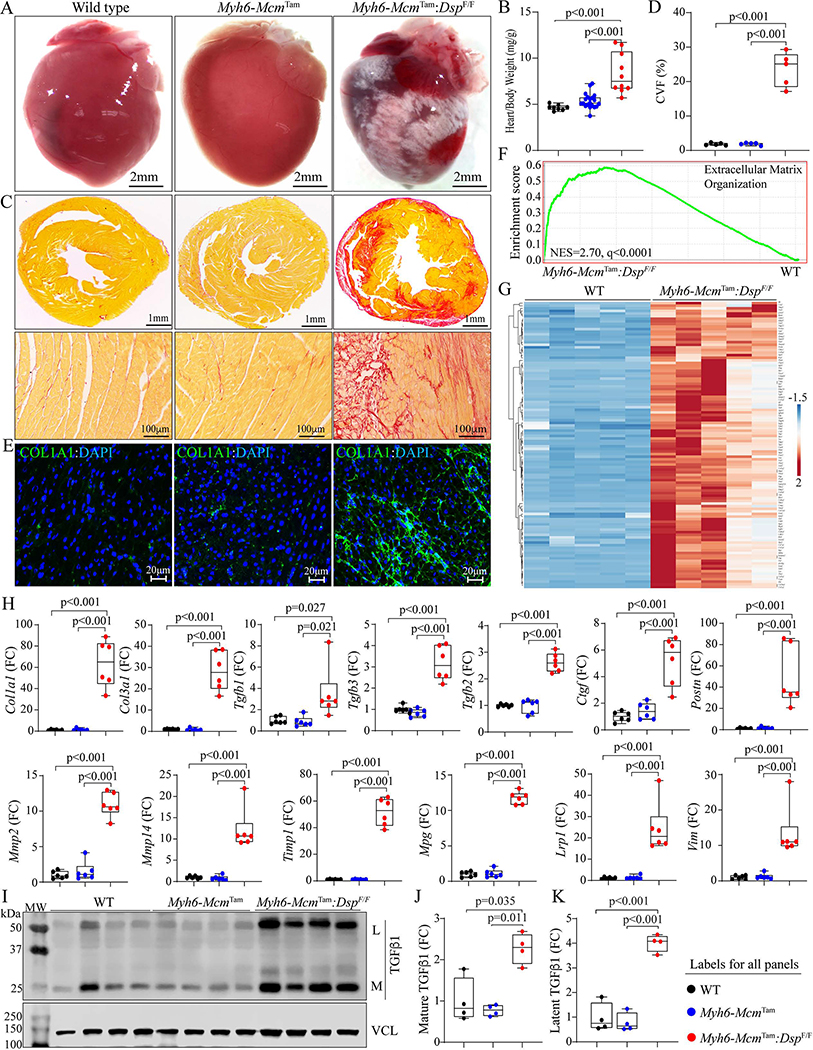
Myocardial fibrosis in the *Myh6-Mcm*^Tam^:*Dsp*^F/F^ mice. (A) Gross heart morphology showing epicardial fibrosis in the *Myh6*-*Mcm*^Tam^:*Dsp*^F/F^ heart. (B) Heart weight/body weight ratio. (C) Low (upper panel) and high (lower panel) magnification of thin myocardial sections stained with picrosirius red showing extensive myocardial fibrosis (red). (D) Quantitative data of myocardial fibrosis presented as collagen volume fraction as the percentage of myocardium occupied by fibrosis. (E) Immunofluorescence staining of thin myocardial sections for COL1A1 protein, a marker of fibrosis. (F) GSEA plot showing enrichment of genes involved in extracellular matrix organization. (G) Heat map of the DEGs showing upregulation of genes involved in fibrosis in the *Myh6-Mcm*^Tam^:*Dsp*^F/F^ heart. (H) Dot plots depicting transcript levels of over a dozen genes involved in fibrosis in the experimental groups. (I) Immunoblot showing expressing of the latent and mature TGFβalong with that of VCL, the latter as a loading control. (J) Dot plots showing quantitative data of the mature TGFβ protein levels in the cardiac protein extracts from the experimental groups. (K) Dot plots showing quantitative data of the latent TGFβ protein levels, as in panel J.

A striking feature of the gross cardiac morphology was marked epicardial fibrosis involving both ventricles, without a clear predilection toward the involvement of a specific chamber or myocardial region [Figure 5A]. Consistent with severe epicardial fibrosis and cardiac dysfunction, the HW/BW ratio was markedly increased in the 4 to 6-week-old *Myh6-Mcm*^Tam^:*Dsp*^F/F^ compared to the WT mice [Figure 5B]. Staining of the thin myocardial sections with picrosirius red showed markedly increased CVF, which comprised approximately 25% of the myocardium, compared to the WT mice, whereas no significant change in the CVF was detected in the *Myh6-Mcm*^Tam^ mice [Figure 5C and D]. Similarly, the staining of thin myocardial sections with an antibody against COL1A1 protein showed patchy areas of an intense fluorescence signal [Figure 5E].

GSEA of the RNA-Seq data showed significant enrichment of genes involved in the extracellular matrix organization in the *Myh6-Mcm*^Tam^:*Dsp*^F/F^ compared to the WT myocytes [Figure 5F]. A heat map of over 100 DEGs involved in EMT showed increased transcript levels of the DEGs in the *Myh6-Mcm*^Tam^:*Dsp*^F/F^ myocytes [Figure 5G]. Transcript levels of over a dozen genes involved in myocardial fibrosis, namely *Col1a1*, *Col3a1*, *Tgfb1*, *Tgfb2*, *Tgfb3, Ctgf*, *Postn*, *Mmp2*, *Mmp14*, *Timp1*, *Mgp*, *Lrp1*, and *Vim* were analyzed by RT-PCR in independent samples from those used in the RNA-Seq, which showed marked increase in the levels of all selected genes in the *Myh6-Mcm*^Tam^:*Dsp*^F/F^ myocytes compared to the corresponding levels in the WT or *Myh6-Mcm* mice [Figure 5H]. Analysis of the myocardial protein extracts for the expression of the TGFβ 1 protein, a well-established pro-fibrotic mitotic factor, by immunoblotting showed increased levels of the latent and mature forms of the TGFβ1 protein in the *Myh6-Mcm*^Tam^:*Dsp*^F/F^ hearts [Figure 5I–K].

### Myocardial adiposis:

There was no significant change in the number of adipocytes in the myocardium, as detected upon staining of thin myocardial sections with PLIN1, which tags triglyceride-rich mature adipocytes [Supplementary Figure 4A and B]. Transcript levels of several genes involved in adipogenesis were analyzed by RT-PCR. The findings were notable for increased transcript levels of adipogenic transcription factors CEBPA and PPARG; however, overall, there was no consistent pattern in the expression of genes involved in adipogenesis [Supplementary Figure 4C].

### Cardiac size and function:

**Figure 6.**
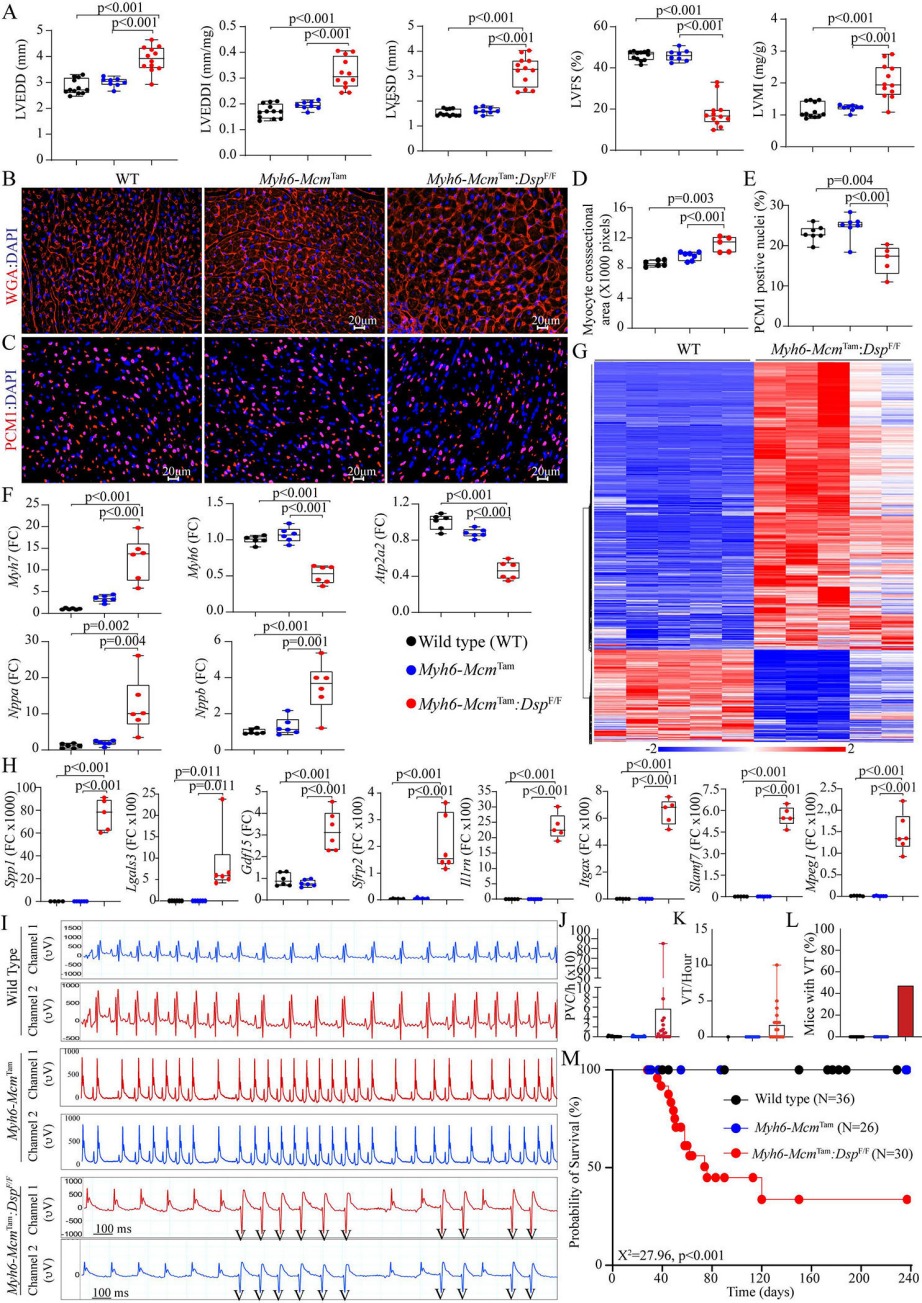
Cardiac dysfunction, arrhythmias, and survival in the *Myh6-Mcm*^Tam^:*Dsp*^F/F^ mice. (A) Dot plots depicting selected indices of cardiac size and function. (B) Immunofluorescence panels showing wheat germ agglutinin (WGA) stained thin myocardial sections. (C) Immunofluorescence panels showing thin myocardial sections stained with an antibody against pericentriolar material 1 (PCM1) to mark myocyte nuclei in the heart. (D) Dot plots representing myocyte cross-sectional areas in the experimental groups, calculated from the WGA-stained sections and corrected for the number of PCM1-tagged myocytes. (E) Dot plots depicting the number of PCM1-tagged myocytes in the experimental groups. (F) Dot plots showing transcript levels of selected markers of cardiac hypertrophy and failure. (G) Heat map depicting DEGs whose protein products are expected to be secreted (secretome), illustrating upregulation of most genes coding for the secretome in the *Myh6-Mcm*^Tam^:*Dsp*^F/F^ cardiac myocytes. (H) Dot plots showing transcript levels of several genes encoding the secretome in the cardiac myocytes in the experimental groups. (I) Two-lead surface electrocardiographic strips showing premature ventricular complexes (PVCs) and runs of non-sustained ventricular tachycardia (NSVT) in the *Myh6-Mcm*^Tam^:*Dsp*^F/F^ mice. (J) Dot plots depicting the number of PVCs per hour detected in the WT, *Myh6-Mcm* ^Tam^, and *Myh6-Mcm*^Tam^:*Dsp*^F/F^ mice. (K) Dot plots showing the number of NSVT episodes in the experimental groups. (L) Bar graph showing the number of mice showing NSVT episodes. (M) Kaplan-Meier survival plots in the experimental groups. Each circle identified one mouse alive at that point and each vertical drop indicated a death.

The *Myh6-Mcm*^Tam^:*Dsp*^F/F^ mice exhibited severe cardiac dilatation and dysfunction at 4 weeks of age, consistent with their high mortality rate [Table 1]. Selective indices of left ventricular size and function are presented in Figure 6A, indicating increased LVEDD, LVESD, and LVMI and markedly reduced LVFS. There were no sex-dependent differences in the echocardiographic indexes. To further assess the increased LVMI in the *Myh6-Mcm*^Tam^:*Dsp*^F/F^ mice, cardiac myocyte CSA was determined upon WGA and PCM1 co-staining of the myocardial sections [Figure 6B and C]. The former was used to quantify the size of the interstitial tissue and the latter to calculate the number of myocytes. Quantitative data showed that cardiac myocyte CSA was increased in the *Myh6-Mcm*^Tam^:*Dsp*^F/F^ mice, compared to the WT or *Myh6-Mcm*^Tam^ mice [Figure 6D]. In contrast, the number of cardiac myocytes, identified as myocardial cells expressing PCM1, was reduced in the *Myh6-Mcm*^Tam^:*Dsp*^F/F^ mouse hearts [Figure 6E]. Moreover, transcript levels of markers of cardiac hypertrophy and dysfunction were assessed by RT-PCR in the isolated myocytes, which showed increased transcript levels of *Nppa*, *Nppb*, and *Myh7* and decreased levels of *Myh6* and *Atp2a2* transcripts, all consistent with cardiac hypertrophy and dysfunction [Figure 6F]. Furthermore, the RNA-Seq data were analyzed for the differential expression of genes whose encoded proteins are considered secreted proteins (secretome). There was a total of 957 DEGs encoding the secretome in the dataset, of which 725 were up- and 232 were down-regulated, as depicted in the heat map, in the *Myh6-Mcm*^Tam^:*Dsp*^F/F^ myocytes [Figure 6G]. Transcript levels of several secreted biomarkers of heart failure were quantified by RT-PCR and were increased in the *Myh6-Mcm*^Tam^:*Dsp*^F/F^ cardiac myocytes [Figure 6H].

### Cardiac arrhythmias:

Deficiency of the *Dsp* gene (*Myh6-Mcm*^Tam^:*Dsp*^F/F^) was associated with a significant increase in the prevalence of ventricular arrhythmias, including premature ventricular contractions (PVCs) and runs of ventricular tachycardia (VT), whereas ventricular arrhythmias were absent in the WT and *Myh6-Mcm*^Tam^ mice [Figure 6I–L]. The number of PVCs per hour was markedly increased, albeit with a wide intra-genotype variation, in the *Myh6-Mcm*^Tam^:*Dsp*^F/F^ mice [Figure 6J]. Likewise, the average number of VT episodes per hour varied from 2 to 325 episodes in the *Myh6-Mcm*^Tam^:*Dsp*^F/F^ mice, while VT was not detected in the WT or *Myh6-Mcm*^Tam^ mice [Figure 6K]. Four out of 15 *Myh6-Mcm*^Tam^:*Dsp*^F/F^ mice developed VT episodes during one hour of cardiac rhythm monitoring, but none of the 14 WT or 13 *Myh6-Mcm*^Tam^ mice showed VT episodes [Figure 6L].

### Survival:

Homozygous deletion of the *Dsp* gene in the post-natal myocytes was associated with a high rate of premature death, as indicated by the premature death of ~2/3rd of the *Myh6-Mcm*^Tam^:*Dsp*^F/F^ mice by 6 months of age [Figure 6M]. There was no sex-dependent difference in the survival rates. The *Myh6-Mcm*^Tam^ mice exhibited a normal survival of up to 8 months^[28]^.

## DISCUSSION

Cell death and myocardial fibrosis are the cardinal features of heart failure and hereditary cardiomyopathies, particularly ACM caused by mutations in the human *DSP* gene^[16,20]^. Deletion of the *Dsp* gene in the post-natal cardiac myocytes led to the extensive molecular remodeling of the IDs, characterized by the post-transcriptional reduction of levels of the desmosome proteins and activation of the receptor- and mitochondrial-mediated cell death programs, including apoptosis, necroptosis, and pyroptosis, i.e., PANoptosis. Consistent with the activation of PANoptosis programs, the expression level of ZBP1 protein, which is considered a “master” regulator of PANoptosis, was up-regulated^[42]^. In conjunction with the prominence of cell death, there was extensive myocardial as well as epicardial fibrosis, comprising ~25% of the myocardium, along with increased expression of markers of pro-inflammatory genes. Consequently, the cardiac systolic function was severely depressed, ventricular arrhythmias were common, and survival was markedly diminished. The model, which captures the essential phenotype of human DSP cardiomyopathy, is expected to decipher the molecular mechanisms involved in the pathogenesis of this unique form of ACM.

The phenotype in the *Myh6-Mcm*^Tam^:*Dsp*^F/F^ mouse model resembles the phenotype of DSP-related cardiomyopathy, which is characterized by the prominence of cell death, inflammation, myocardial fibrosis, left-dominant or biventricular cardiomyopathy, and progressive heart failure^[16,18–20]^. The *Dsp* null genotype, which was induced specifically in the post-natal myocytes, represents the recessive and compound mutations in the human *DSP* gene, which are established causes of ACM and the cardiocutaneous syndromes characterized by DCM, woolly hair, and keratoderma^[14,43,44]^. Given that the *Dsp* gene was specifically deleted in the post-natal cardiac myocytes but not in other cell types, the phenotype was restricted to the heart and there was no cutaneous component. The model represents the phenotypic extreme as opposed to the heterozygous *Myh6-Mcm*^Tam^:*Dsp*^W/F^ mice, which show a milder and slowly evolving phenotype largely similar to that in the published models^[22,29]^.

The prominence of cell death in the *Myh6-Mcm*^Tam^:*Dsp*^F/F^ mice was notable for the activation of PANoptosis, as indicated by changes in the markers of apoptosis, necroptosis, and pyroptosis. Moreover, the RNA-Seq data supported the activation of the cell death programs through the tumor necrosis factor receptor (TNFR) superfamily and increased expression of the pro-inflammatory genes through the NFκB pathway. To our knowledge, this is the first report of PANoptosis in the heart. Whether PANoptosis is a common feature and plays an important role in the pathogenesis of myocardial diseases, including other forms of hereditary cardiomyopathies and heart failure, is an empiric question worth testing.

The initial impetus of increased cell death in the *Myh6-Mcm*^Tam^:*Dsp*^F/F^ mice and how the molecular remodeling of the IDs activates the cell death programs remain to be delineated. The transcriptomic data suggest activation of the TNFR superfamily and death receptor-mediated pathways in the *Myh6-Mcm*^Tam^:*Dsp*^F/F^ myocytes. Whether altered mechanical properties of the cell membrane initiates the cell death programs and/or whether altered molecular interactions between the constituents of the remodeled IDs and death receptor pathway molecules instigate the activation of the cell death programs remains to be determined. It is also plausible that altered mechano-sensitive signaling pathways, such as the Hippo pathway and the integrins and the downstream effectors, such as the EP300 and TP53 pathway, provide the initial impetus for cell death^[22–24,45]^. Moreover, CASP3 and cell death receptor molecules TRAF4, TNIK, DR4, and DR5 are known to interact with the ID proteins and might be activated in response to the molecular remodeling of the IDs in the *Myh6-Mcm*^Tam^:*Dsp*^F/F^ myocytes^[46–49]^. Initiation of cell death, regardless of the primary underpinning mechanism, could lead to the release of damage-associated molecular patterns (DAMPs), triggering the innate immune system, activating inflammation, and perpetuating cell death by pyroptosis. It is intriguing that inflammation has been recognized as an important phenotypic component of ACM, which might be explained, at least in part, by the activation of the cell death programs^[50]^.

Myocardial fibrosis, assessed by determining CVF, expression levels of a dozen genes involved in fibrosis, and activation of the TGFβ1, was extensive, comprising about 25% of the myocardium, a finding that is in accord with the pro-fibrotic cardiomyopathy caused by the *DSP* mutations in humans^[16,20]^. Myocardial fibrosis is likely secondary to the expression of the pro-fibrotic trophic and mitotic factors, such as TGFβ1 from the cardiac myocytes, which is also observed in other forms of hereditary cardiomyopathy^[38,51]^. In addition, myocyte death is also expected to contribute to myocardial fibrosis, which is often referred to as replacement fibrosis. Overall, the extent of myocardial fibrosis is severe and, by and large unusual among the mouse models of hereditary cardiomyopathies, rendering the model desirable for mechanistic studies and therapeutic targeting of myocardial fibrosis.

The efficiency of the tamoxifen-inducible Cre-mediated deletion of the *Dsp* gene in the post-natal was evident by the near complete absence of the DSP protein in the myocardial protein extracts and at the IDs. It is also noteworthy that levels of several other desmosome proteins, namely JUP, PKP2, DSC2, and DSG2, all implicated as causes of ACM, were markedly reduced. This finding is in accord with the previous data, including data in humans, and has been attributed to the activation of the proteolytic pathways in ACM^[24,25]^. Reduced expression and localization of multiple desmosome proteins are in part responsible for the severity of the phenotype observed in this model.

The study has several limitations. As discussed earlier, the precise mechanism(s) by which DSP deficiency leads to PANoptosis is unclear. Likewise, the mechanism(s) responsible for the increased expression of ZBP1 and whether upregulation of the ZBP1 protein is solely or partly responsible for PANoptosis in the present model is unknown. Moreover, the findings are restricted to a single mouse model of ACM, whereby the *Dsp* gene was almost completely deleted. Although homozygous DSP mutations are known causes of human ACM, most of the mutations causing ACM are heterozygous mutations^[14,43,44]^. Thus, the relevance of the findings to the broad category of ACM or DCM is unclear. The *Dsp* gene was deleted conditionally at the post-natal day 14, in contrast to the humans where the mutation is present since single-cell embryo formation. The approach was taken to avoid the embryonic lethality associated with homozygous deletion of the *Dsp* gene and because of the slow evolution of the phenotype in mice with the heterozygous deletion of the *Dsp* gene^[21,22,29]^. Finally, injection of tamoxifen and induction of activation of the MerCreMer fusion protein is associated with transient cardiac dysfunction^[52,53]^. To reduce such confounding effects, phenotypic characterization was performed 2 weeks after initiation of the Cre-mediated recombination, and a lower dose of tamoxifen was used^[54]^. In addition, genes whose expressions are affected by the injection of tamoxifen and the expression of MerCreMer were excluded from the analysis^[28]^.

In conclusion, post-natal homozygous deletion of the *Dsp* gene caused severe early-onset DCM characterized by fulminant PANoptosis, severe fibrosis, and increased expression of the pro-inflammatory genes, which are the phenotypic features of DSP cardiomyopathy. The findings set the stage for subsequent mechanistic studies to delineate the molecular pathogenesis of DSP cardiomyopathy. The model also provides the opportunity to test the effects of pharmacological and genetic interventions in preventing or attenuating cell death, fibrosis, and inflammation in DSP cardiomyopathy.

## Supplementary Material

Supplementary Material

## Figures and Tables

**Table 1.** Echocardiographic measurements of cardiac size and function in the wild type (WT), *Myh6-Mcm*^Tam^ and *Myh6-Mcm*^Tam^*:Dsp*^F/F^ mice

	WT	*Myh6-Mcm* ^Tam^	*Myh6-Mcm*^Tam^:*Dsp*^F/F^	One way ANOVA

N	12	8	12	NA
M/F	6/6	4/4	6/6	0.961*
Age (days)	28.17 ± 0.41	28.50 ± 0.53	28.08 ± 0.29	0.081^#^
Body weight (g)	18.40 ± 1.76	15.55 ± 0.92	12.45 ± 1.50	< 0.0001
HR (bpm)	664.90 ± 25.98	673.99 ± 36.93	591.06 ± 40.83	< 0.0001
AWT(mm)	0.39 ± 0.02	0.34 ± 0.02	0.29 ± 0.03	< 0.0001
LVPWT(mm)	0.38 ± 0.02	0.34 ± 0.02	0.28 ± 0.03	< 0.0001
LVEDD (mm)	2.85 ± 0.28	3.01 ± 0.18	3.91 ± 0.49	< 0.0001^#^
LVEDDI (mm/g)	0.16 ± 0.03	0.19 ± 0.02	0.32 ± 0.06	< 0.0001^#^
LVESD (mm)	1.54 ± 0.15	1.62 ± 0.13	3.21 ± 0.58	< 0.0001^#^
LVFS (%)	46.05 ± 1.81	46.10 ± 2.77	18.25 ± 7.12	< 0.0001^#^
LV Mass (mg)	20.46 ± 4.21	18.94 ± 1.97	25.24 ± 5.99	0.009^#^
LVMI (mg/g)	1.12 ± 0.26	1.22 ± 0.10	2.05 ± 0.55	0.0001^#^

*Myh6-Mcm:* Myosin heavy chain 6-MerCreMer; *Dsp:* desmoplakin gene; M/F: male/ Female; g: grams; HR: heart rate; bpm: beats per minute; AWST: anterior wall thickness; LVPWT: left ventricular posterior wall thickness; LVEDD: left ventricular end-diastolic diameter; mm: millimeters; LVEDDI: LVEDD indexed to body weight; LVESD: left ventricular end-systolic diameter; LVFS: left ventricular fractional shortening; LVM: left ventricular mass; LVMI: LVM indexed to body weight.

* Denotes *P*-value obtained by chi-square test

# Denotes *P*-value obtained by Kruskal- Wallis test; Data in the WT and *Myh6-Mcm* ^Tam^, in part, include mice reported in the previous studies^[28,35]^.

## Data Availability

RNA-Seq data have been submitted to GEO and are available to the public upon release (GSE180972).
