# Supplementary material for: PANoptosis is a prominent feature of desmoplakin cardiomyopathy
Source: J Cardiovasc Aging. Author manuscript; Available in PMC 2023 Feb 16. (PMC9933912; doi:10.20517/jca.2022.34)
Supplement: Supplementary Material [file NIHMS1846283-supplement-Supplementary_Material.pdf]

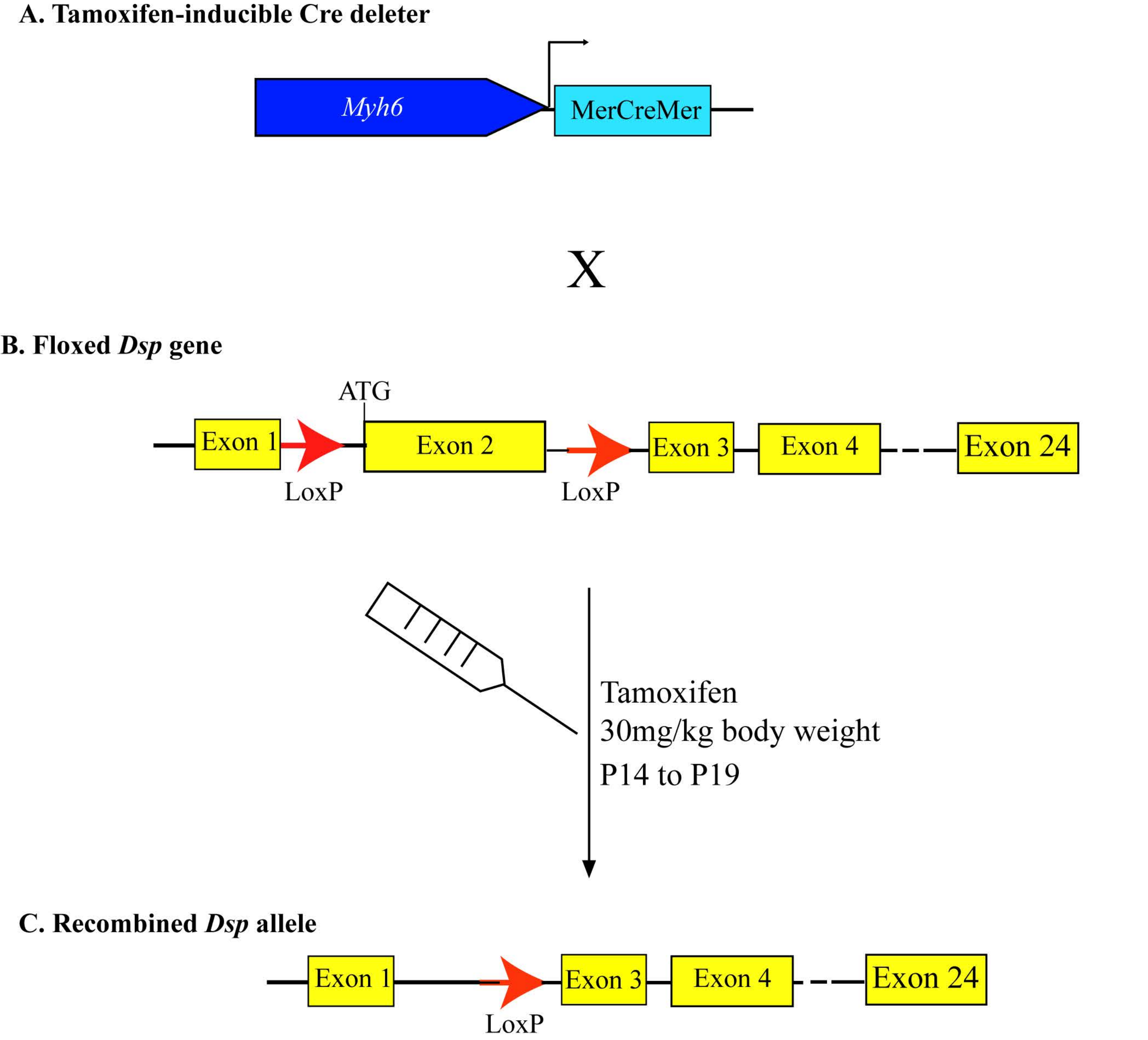

Online Figure 1. Schematic illustration of deletion of the *Dsp* gene in cardioac myocyte post-natally
A. Cardiac myocyte-specific tamoxifen-inducible cre deleter mice
B. Floxed *Dsp* gene wherein exon 2, containing the initiation codon, is floxed by the LoxP sequences
C. The recombined *Dsp* allele after deletion of exon 2

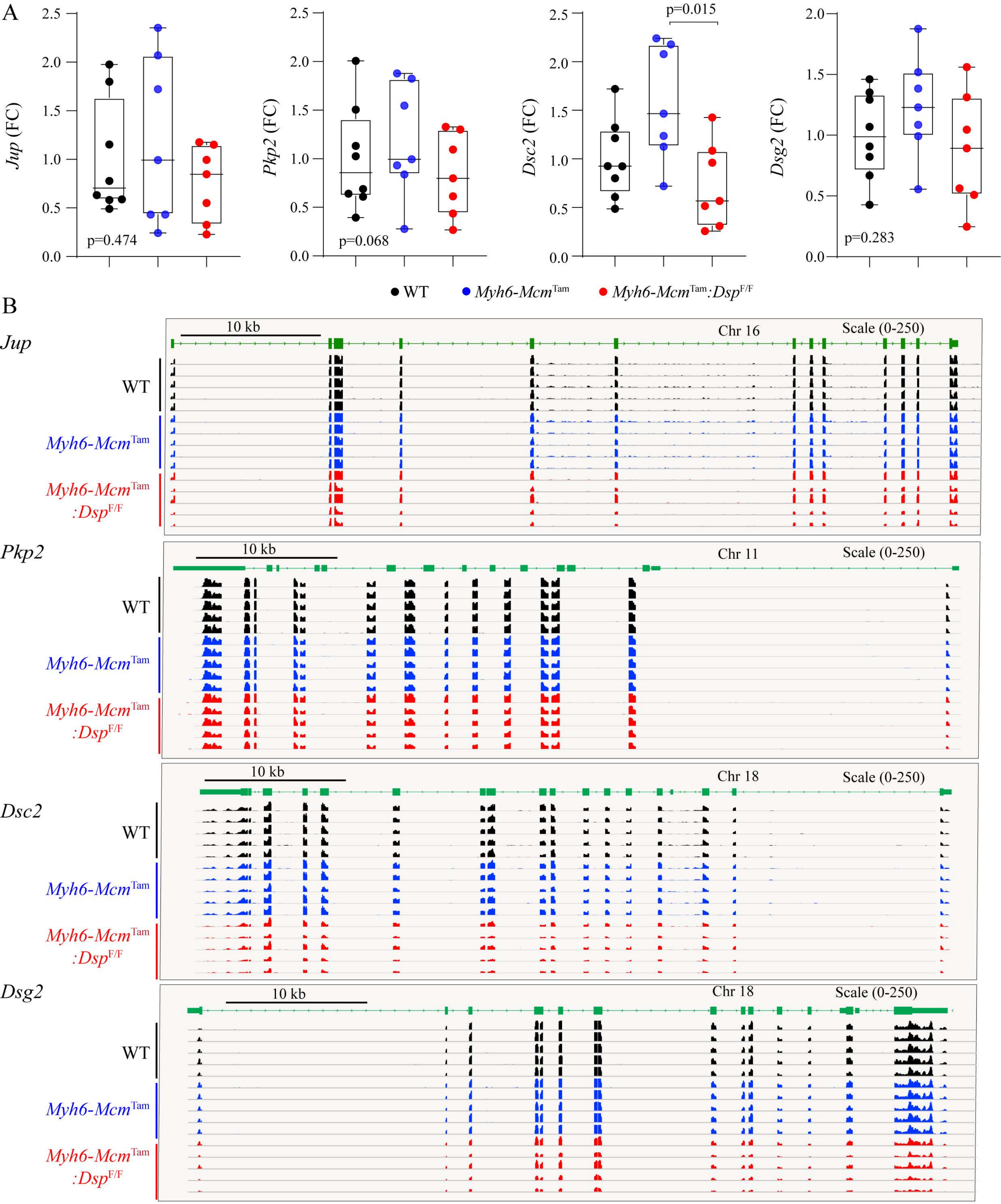

Online Figure 2. Transcript levels and genome browser maps of selected genes encoding desmosome proteins.

A. RT-PCR data showing transcript levels of the selected genes among the three genotypes, which were unchanged.

B.The genome browser depiction of the the RNA-Seq reads mapped of the exons of the selected desmosome genes in the wild type (WT), *Myh6-Mcm*^{Tam}, and the *Myh6-Mcm*^{Tam}-*Dsp*$^{F/F}$ groups.

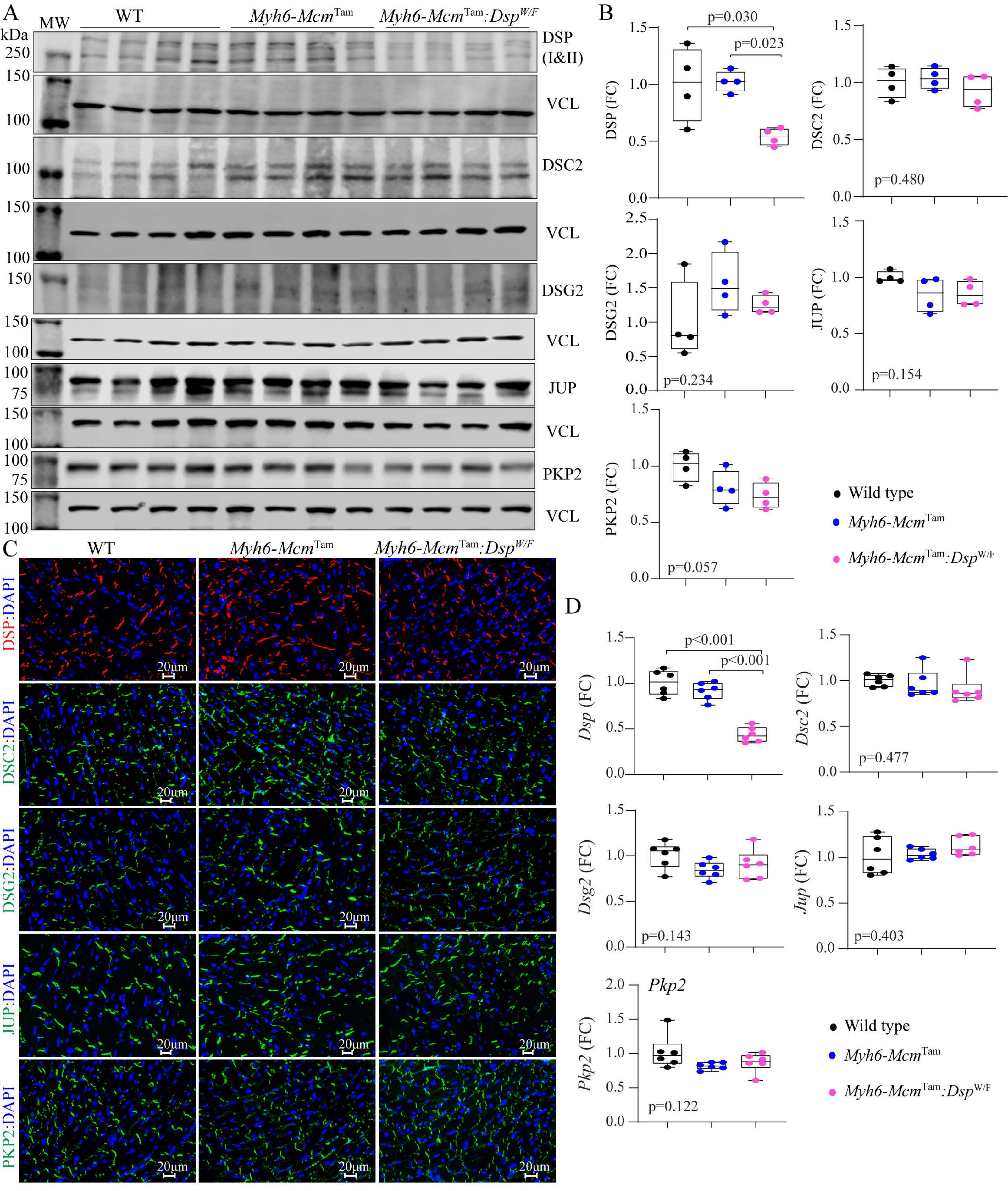

Online Figure 3. Heterozygous deletion of the *Dsp* gene in the post-natal cardiac myocytes.

A and B. Expression levels of the DSP and selected desmosome proteins in cardiac myocytes.

C. Expression and localization of the DSP and selected desmosome proteins to the intercalated discs in the experimental groups.

D. Transcript levels of the *Dsp* and selected desmosome genes are depicted. Only transcript levels of *Dsp* were reduced in accord with the genetic detetion of one copy of the *Dsp* gene.

Abbreviations: WT: wild type, FC: fold change

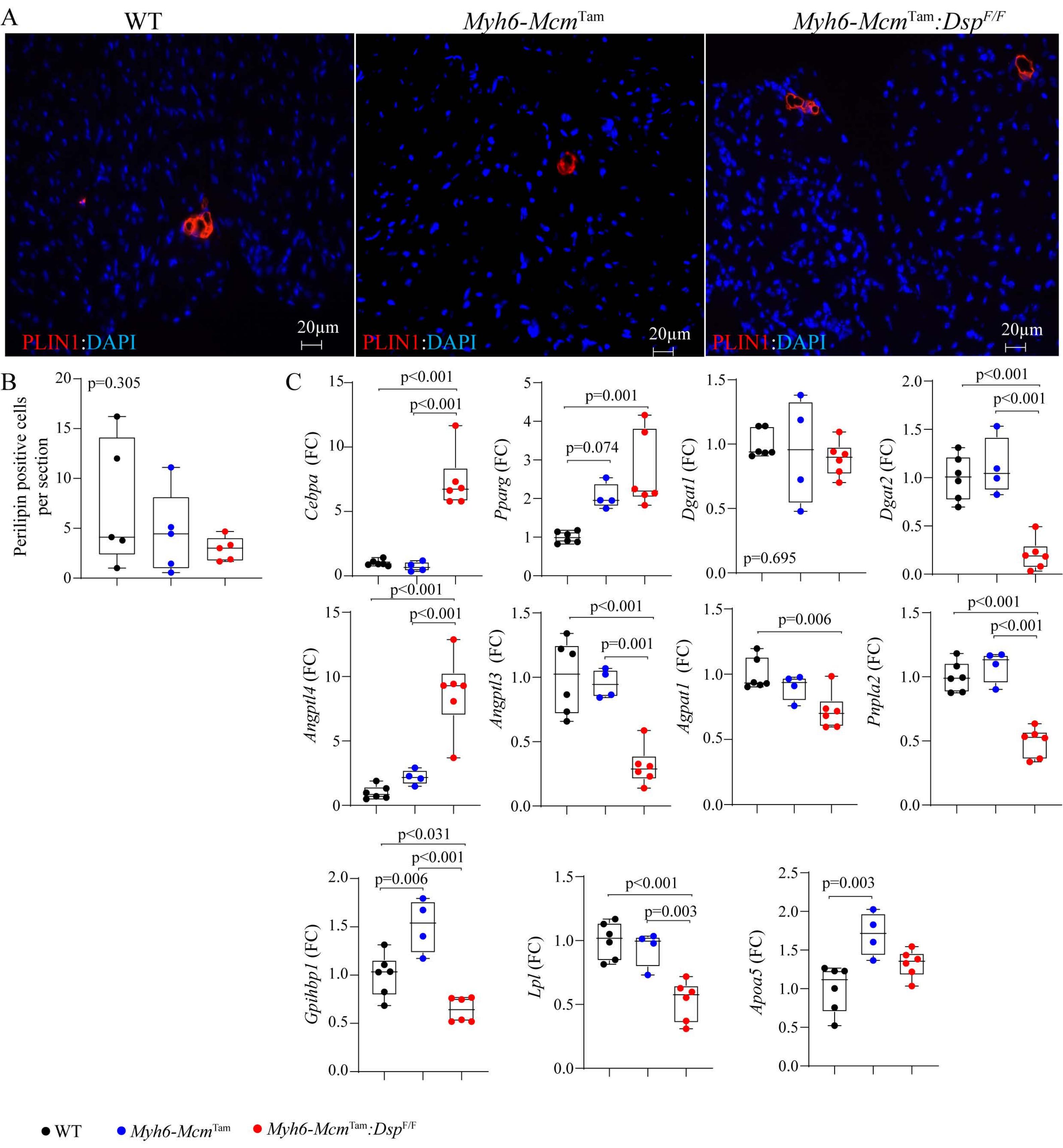

Online Figure 4. Adipogenic markers in the heart of *Myh6-Mcm*^{Tam}*:Dsp*$^{F/F}$ mice.

A. Thin myocardial sections stained with perlipin1 (PLIN1), a marker of mature adipocytes.

B. Quantitative data showing the mean number of PLIN1-stained adipocytes per section in the control and experimental groups.

C. Dot plots showing the RT-PCR data representing transcript levels of selected genes involved in adipogenesis.

Online Table 1: Oligonucleotide Primers, Antibodies, and TaqMan probes

Genotyping primers:

Gene	Forward sequence	Reverse sequence
Cre recombinase	TCTATTGCACACAGCAATCCA	CCAGCATTGTGAGAACAAGG
Desmoplakin	GTTGGGCCTCTCGAATCAT	TCTTTGTCTGTTGCCATGTGA

List of antibodies

Antibodies	Concentration	Supplier	Catalog number
Anti-mouse IgG HRP linked antibody	1:4000 (IB)	Cell Signaling Technology	7076
Anti-Rabbit IgG HRP linked antibody	1:2000 (IB)	Cell Signaling Technology	7074
ASC	1:1000 (IB)	Cell Signaling Technology	67824
BAD	1:500 (IB)	Santa Cruz	8044
BCL2	1:500 (IB)	Santa Cruz	7382
CASP3	1:1000 (IB)	Cell Signaling Technology	14220
CASP8	1:1000 (IB)	Cell Signaling Technology	4927
COL1A1	1:300 (IF)	Abcam	88147
Donkey anti Rat Alexa Fluor 594	1:1000 (IF)	Invitrogen	A11007
DSC2	1:200 (IB)	Santa Cruz	70994
DSC2	1:100 (IF)	Santa Cruz	66863
DSG2	1:500 (IB)	Progen	61002
DSG2	1:50 (IF)	Progen	61002
DSP	1:1000 (IB)	Abcam	ab71690
DSP	1:250 (IF)	Progen	65146
Goat anti Mouse Alexa Fluor594	1:1000 (IF)	Invitrogen	A11005
Goat anti Rabbit Alexa Fluor 488	1:1000 (IF)	Invitrogen	A11012
GSDMD	1:1000 (IB)	Cell Signaling Technology	39754
HMGB1	1:500 (IB)	Cell Signaling Technology	6893
JUP	1:1000 (IB)	Santa Cruz	1497-R
JUP	1:1000 (IF)	Santa Cruz	1497-R
MLKL	1:1000 (IB)	Cell Signaling Technology	37705
PCM1	1:1000 (IF)	Sigma	HPA023370
PCM1	1:50 (IF)	Santa Cruz	Sc-398365
PKP2	1:1000 (IB)	Santa Cruz	18977
PKP2	1:300 (IF)	Progen	651101
PLIN	1:100 (IF)	Cell Signaling Technology	9349
RIPK1	1:1000 (IB)	Cell Signaling Technology	3493
RIPK3	1:1000 (IB)	Cell Signaling Technology	15828
TGFB	1:500 (IB)	R&D Systems	MAB1835
TUBA1A	1:1000 (IB)	Cell Signaling Technology	2125
Vinculin	1:10000 (IB)	Abcam	Ab129002
ZBP1	1:500 (IB)	Santa Cruz	271483

Oligonucleotide primers used in qPCR reactions

Name	Sequence
Agpat1	Forward: AACCTTTGTCGGTTCCGCT<br>Reverse: GACAGAGATACAGCCAGCCG
Angptl3	Forward: TTTGGGAGGCTCGATGGAGAA<br>Reverse: TGCTTGCTGTCTTTCCAGTCT
Angptl4	Forward: GACTGCCAGGAACTCTTCCAA<br>Reverse: GTCCAGCCTCCATCTGAAGT
Apoa5	Forward: AACCGAGCAGGGGCCAT<br>Reverse: TAGTCCCAGAGGCTCTTCCG
Asc	Forward: GAGCAGCTGCAAACGACTAA<br>Reverse: GACCCTGGCAATGAGTGCTT
Bak	Forward: CAAGATCGCCTCCAGCCTATT<br>Reverse: CCCAGGAAGCCGGTCAAAC
Bax	Forward: ACAGGGGCCTTTTTGCTACA<br>Reverse: CACTCGCTCAGCTTCTTGGT
Bid	Forward: CCGCAAACCTTTGCCTTAGC<br>Reverse: AACCGTTGCTGACCTCAGAGT
Bok	Forward: CAGCGTATACCGGAACGTGG<br>Reverse: TTGCCCCATGTGATACCTGC
Casp1	Forward: ACTGACTGGGACCCTCAAGT<br>Reverse: GCAAGACGTGTACGAGTGGT
Casp3	Forward: AGCTTGGAACGGTACGCTAA<br>Reverse: GAGTCCACTGACTTGCTCCC
Casp8	Forward: GCGTGGAACAGGAAGTGAGTA<br>Reverse: GAAGAGCTGTAACCTGTGGC
Col1a1	Forward: TGACTGGAAGAGCGGAGAGTA<br>Reverse: GAGTAGGGAACACACAGGTCT
Col3a1	Forward: CTGTAACATGGAAACTGGGGAAA<br>Reverse: CCATAGCTGAACTGAAAACCACC
Gdf15	Forward: CTCAACGCCGACGAGTAC<br>Reverse: ACCCCAATCTCACCTCTGGA
Gpihbp1	Forward: CACAGCGGAACCGACAAAG<br>Reverse: GACTGGCAACAGGTCTGAGT
Gsdmd	Forward: TTCCAGTGCCTCCATGAATGT<br>Reverse: ACAAACAGGTCATCCCCACG
Il1rn	Forward: ATCCTTCTGTTTCATTCAGAGGC<br>Reverse: TCCCAGATTCTGAAGGCTTGC
Itgax	Forward: TACGTATTCAGCGTGGAGAACTT<br>Reverse: TGAAGCCCTCCTGGGACATC
Lgals3	Forward: TACTAGAAGCGGCCGAGC<br>Reverse: TGTCTGCCATTTTCCTGGGTA
Lpl	Forward: TACCCCCTAGACAACGTCCA<br>Reverse: CCAGCTGGATCCAAACCAGT
Lrp1	Forward: CAAAGCTGAAGGCTCCGAGT<br>Reverse: TATGCGGACACTCTCATCGC

Mgp	Forward: GCAACCCTGTGCTACGAATC<br>Reverse: CTTTTGGGCTTTAGCTCGCC
Mlkl	Forward: GTATTCAACAACCCCCAGGC<br>Reverse: CGCAAGATGTTGGGAGAATCG
Mmp14	Forward: GCCCTCTGTCCCAGATAAGC<br>Reverse: CCAGAACCATCGCTCCTTGA
Mmp2	Forward: TGCCCCCATGAAGCCTTG<br>Reverse: TGGTACAGCTGTTGTAGGAGG
Mpeg1	Forward: ACAAAAGCCAGACAGAGCCTT<br>Reverse: AGGATGGAAAAGAAAGCGGTTG
Noxa	Forward: ATAACTGTGGTTCTGGCGCA<br>Reverse: CAATCCTCCGGAGTTGAGCA
Pnpla2	Forward: AATCTCTACCGCCTCTCGAA<br>Reverse: AGGGTTGGGTTGGTTCAGTA
Postn	Forward: AGAGAAATCCCTGCACGACA<br>Reverse: GTTGGTGCAAACAAGGTCCA
Ripk1	Forward: TGACTTTCACATTAAGATAGCCGA<br>Reverse: AGTGGTGCTGCTCACTTCTTT
Ripk3	Forward: AGCTTTGGGATCCTCGTGTG<br>Reverse: TCTGTCAGTGGAGGACGACT
Sfrp2	Forward: CCCTTTGTAAAAATGACTTCGCAC<br>Reverse: CAGGATGATCTTGGTGTCTCTGT
Slamf7	Forward: GACTGACGACCTGAAAACGC<br>Reverse: CGGCCACCTTCTTCAGAGTT
Spp1	Forward: AGCAAGAAACTCTTCCAAGCAA<br>Reverse: GTGAGATTCGTCAGATTCATCCG
Tgfb1	Forward: TGGAGCAACATGTGGAACTC<br>Reverse: GTCAGCAGCCGGTTACCA
Tgfb2	Forward: AGGAGTGGCTTCACCACAAAGACA<br>Reverse: ATTAGACGGCACGAAGGTACAGCA
Tgfb3	Forward: AGCTCTTCCAGATACTTCGACC<br>Reverse: AAAGACAGCCATTCAGCGGT
Timp1	Forward: CATGGAAAGCCTCTGTGGATA<br>Reverse: CTCAGAGTACGCCAGGGAAC
Tnfrsf10b/Dr5	Forward: TCGTCTCTCTTGGACCGACA<br>Reverse: GTCAGCTGAGTCGTTTCCGT
Tnfrsf19/Troy	Forward: AGTTGTCCAAGGAATGTGGCT<br>Reverse: CCGCACATGGCTTACACTTC
Traf4	Forward: AGGTCCAGGTGTTAGGCTTG<br>Reverse: GCTGCAAGTGTTCAAGTGGC
Traf6	Forward: CATAAGGGATGCAGGGCACA<br>Reverse: TTGGGCACTTTACCGTCAGG
Vcl	Forward: GGTCTAGCAAGGGCAATGAC<br>Reverse: TGAATAAGTGCCCGCTTGGT
Vim	Forward: TGCACGATGAAGAGATCCAGG<br>Reverse: CTTTCATACTGCTGGCGCAC
Zbp1 ex2-3	Forward: GAGATGGGGCTCCTGCAATC<br>Reverse: CCCATTGGCTTCCAGGAATCTT
Zbp1 ex3-4	Forward: CGTCAGGAAGGCCAAGACAT<br>Reverse: TTGACCGGATTGTGCTGACA

Zbp1 ex4-5	Forward: CAAACGCCATCCAGATTGGTC<br>Reverse: CGTCCCATGCCAAGGGTAGA
Zbp1 ex5-6	Forward: GCCACCACAACGAGATGAGC<br>Reverse: TGTTGAATGAAGCTCCTGGGT

TaqMan probes

Gene	TaqMan Assay ID
Atp2a2	Mm00437634_m1
Cebpa	Mm00514283_s1
Ctgf	Mm01192932-g1
Dgat1	Mm00515643_m1
Dgat2	Mm00499536_m1
Dsc2	Mm00516355_m1
Dsg2	Mm00514608_m1
Dsp	Mm01351876_m1
Jup	Mm00550256_m1
Myh6	Mm00440354_m1
Myh7	Mm0060555_m1
Nppa	Mm01255748_g1
Nppb	Mm01255770_g1
Pkp2	Mm00503159_m1
Pparg	Mm00440940_m1
Vcl	Mm00447745_m1
